# When avoiding failure helps: the interplay of achievement motivations, growth mindset, and career decision-making self-efficacy among pre-service teachers

**DOI:** 10.3389/fpsyg.2026.1842181

**Published:** 2026-05-08

**Authors:** Ming Liu, Ruo-ya Li, Ya-zhou Huang

**Affiliations:** 1 College of Education Science, Northwest Normal University, Lanzhou, China; 2 Faculty of Primary Education, Fuyang Preschool Teachers College, Fuyang, China; 3 School of Education Science, Guangxi Minzu Normal University, Chongzuo, China

**Keywords:** career decision-making self-efficacy, growth mindset, motive to approach success, motive to avoid failure, pre-service teachers

## Abstract

**Introduction:**

Against a context of escalating competition in the teacher labor market and advancing professionalisation in China, it is essential to investigate how the way of achievement motivational orientations jointly influence pre-service teachers’ career decision confidence. This study investigated the mediator of growth mindset between motive to approach success (MS) and career decision-making self-efficacy (CDMSE), and the moderator of motive to avoid failure (MF) between MS and growth mindset.

**Methods:**

Data were collected through convenience sampling from 807 pre-service teachers enrolled at public universities in an eastern province of China, and analyzed utilizing SPSS and SmartPLS.

**Results:**

MS exerted significant positive effects on growth mindset and CDMSE, whereas MF existed a significant negative effect on growth mindset. Growth mindset partially mediated the effect of MS on CDMSE, and MF significantly moderated the path from MS to growth mindset.

**Discussion:**

These results highlight the distinct roles of the two motivational orientations. Educators should not only foster pre-service teachers’ motivation to pursue success but also harness the constructive potential of MF. Rather than being seen as purely negative, MF can encourage reflective thinking and strengthen growth mindset, thereby working in tandem with MS to enhance pre-service teachers’ CDMSE.

## 1 Introduction

China’s higher education system has changed significantly due to both large-scale expansion and structural adjustments in recent years. Although declining birth rates suggest that long-term teacher demand may gradually decrease, the current employment situation for teacher education graduates presents a paradox of ‘numerical surplus alongside qualitative selection’. The number of graduates continues to rise, while the teacher employment market has become increasingly marketised and competitive, with graduates of varying abilities entering the market simultaneously (Dai et al., 2022). This mismatch between supply and quality demand has created occupational uncertainty among pre-service teachers, exacerbating concerns about personal competitiveness and undermining confidence in future teaching careers (Wang et al., 2025).

Previous study has revealed that occupational uncertainty can significantly inhibit career readiness and proactive exploration (Hirschi et al., 2013). Consequently, identifying key psychological resources that can strengthen career confidence in pre-service teachers is both theoretically and practically important. One central personal resource is career decision-making self-efficacy (CDMSE), commonly conceptualized as one’s belief in effectively managing tasks associated with career choices, including self-assessment, gathering occupational information, and selecting career goals (Betz and Luzzo, 1996). Evidence indicates that CDMSE significantly contributes to career adaptability and professional success (Alkal, 2025; Hamzah et al., 2021). Previous evidences also consistently demonstrate that individuals with higher CDMSE engage in systematic information processing and rational evaluation, enabling them to make effective career decisions, while reducing indecision and subsequent regret (Gushue et al., 2006; Prideaux and Creed, 2001).

Moreover, CDMSE serves as a critical psychological buffer against anxiety and stress during the highly uncertain period transitioning from university to the workplace (Park et al., 2019). Given that CDMSE acts as a factor in short-term decision-making quality, long-term career satisfaction, and developmental trajectories (Troesch and Bauer, 2017), examining its formation mechanisms, including its motivational and cognitive antecedents, is theoretically and practically significant.

### 1.1 Motive to approach success, motive to avoid failure, and career decision-making self-efficacy

Bandura (1986) posits self-efficacy is shaped not only formed through past successes and failures, but also by expectations about the future outcomes and perceived control over them. Individuals typically assess what they can achieve and whether effort is worthwhile within a given context by integrating prior experiences and anticipated consequences. This dual information source of experience and outcome expectations shapes generalized self-efficacy and strongly influences career beliefs (Bandura, 1986).

Atkinson (1957) argued that these outcome expectations, or achievement motivation, comprise two different types of achievement motivation:motive to approach success (MS) and motive to avoid failure (MF). MS is directed towards positive outcome expectations, driving individuals to actively engage and pursue success. On the other hand, MF is oriented towards negative outcome expectations, leading individuals to avoid certain situations because of fear of failure and anxiety. Covington (1992) argued that MF does not necessarily mean the absence of MS; rather, it may inhibit or interaction with the positive behavioural expression of MS.

Empirical research confirms that MS is central to career development and decision-making (Glaser, 2012; Joel, 2019). High-MS individuals tend to hold strong expectations of success, which translate into confidence in making effective career decisions, thereby enhancing CDMSE (Glaser, 2012; Tutar et al., 2011). Specifically, when operationalised through career orientations emphasising personal value realisation and growth (Briscoe and Hall, 2006), high-MS university students with stronger career orientations actively seek vocational information and resources, adapt efficiently to evolving economic environments, and demonstrate confidence belief in their career decisions (Herrmann et al., 2015), leading to higher CDMSE (Li et al., 2019). Moreover, MS is highly associated with positive career outcomes. High-MS individuals prefer challenging tasks, and often set ambitious performance goals (Raymark et al., 1997) and strive towards them (Glaser, 2012; Salami, 2004). They also demonstrate greater persistence and resilience through setbacks (Albert et al., 2024; Li et al., 2021) and continuously update their skills preserve competitiveness in the job market (Furness, 2020; Shi, 2022). In contrast, MF, through achievement anxiety, can undermine career decision confidence (Gujral and Bhambri, 2024) and career adaptability (Chuang et al., 2022). Draw on empirical evidence, this research suggests that MS, by driving sustained goal engagement and proactive exploration, promotes higher levels of CDMSE. Although MF may induce anxiety and weaken career confidence, the current research investigates the independent effect of MS on CDMSE, as a key psychological predictor, and hypothesizes that:

*H1*: MS significantly and positively predicts CDMSE.

### 1.2 The mediating role of growth mindset

Career decision-making self-efficacy (CDMSE) represents a key psychological construct shaped by motive to approach success (MS) and motive to avoid failure (MF), illustrating how different outcome expectations influence an individual’s career confidence. Social Cognitive Theory (Bandura, 1986) emphasises that behavioural beliefs are not determined by a single psychological factor; rather, they emerge from multiple interacting cognitive and affective processes. This suggests that there are indirect or mediating pathways through other cognitive mechanisms. MS encourages individuals to interpret challenges and setbacks in a positive, developmental manner, facilitating cognitive restructuring about ability malleability. In contrast, MF heightens threat perception and anxiety, thereby interfering with or inhibiting this process (Heckhausen and Heckhausen, 2018). Within this ‘driving-inhibiting’ dynamic, growth mindset functions as a key latent mechanism linking achievement motivation to CDMSE. However, it is important to note that the interaction between MF and MS may not be strictly antagonistic. Drawing on compensatory control theory (Landau et al., 2015), when the desire to succeed (MS) is coupled with a fear of failure (MF), the resulting psychological tension may increase the urgency for effective coping strategies. In this context, growth mindset might serve as a necessary tool to neutralize the anxiety associated with MF, thereby potentially strengthening the link between MS and growth mindset as an adaptive defensive mechanism.

Social Cognitive Theory further posits that cognitive systems primarily serve to anticipate the potential outcomes of alternative behavioural paths and to regulate behaviour accordingly, thereby enhancing perceived control over important life outcomes (Bandura, 1986). From this perspective, mindset itself may be shaped by outcome expectations, while simultaneously influencing the development of self-efficacy. Dweck (2017) conceptualizes growth mindset referring to the view that one’s capabilities can improve over time with effort and learning, and experience, highlighting the role of effort in achieving better outcomes. A growth mindset leads individuals to approach difficulties as pathways to skill improvement instead of as reflections of personal limitations, and to maintain higher motivation and confidence when facing uncertainties (Chen et al., 2023). This belief that effort leads to skill growth and positive outcomes enables individuals to accumulate successful experiences and efficacy cues during career exploration and decision-making, thereby enhancing CDMSE.

Empirical evidence has shown how growth mindset significantly improves entrepreneurial confidence and vocational development outcomes (Burnette et al., 2020). Individuals with a higher level of growth mindset was linked with higher levels of CDMSE (Park et al., 2022), more proactive involvement in career-related exploration (Huang et al., 2019), reinforcing overall career confidence (Buenconsejo and Datu, 2020). Growth-oriented teachers prioritise student learning and adopt a wider repertoire of instructional approaches within educational settings (Rissanen et al., 2019), which further enhances their teaching self-efficacy, personal achievement, and vocational identity (Zarrinabadi et al., 2023). Similarly, among university populations, stronger growth-oriented beliefs are linked to sustained persistence in career exploration and enhanced confidence in vocational decision (Park et al., 2022). Taken together, growth mindset not only directly enhances CDMSE but may also mediate the effect of achievement motivation. Based on previously empirical evidence, we hypothesised that:

*H2*: Growth mindset positively predicts CDMSE.

According to Social Cognitive Theory (Bandura, 1986), outcome expectations are linked to individuals’ cognitive styles. Outcome expectations not only directly influence motivational orientation but also shape perceptions of ability, challenges, and failure, thereby affecting other key psychological processes (Bandura, 1986). In other words, outcome expectations and mindset do not operate in isolation but interactively shape behavioural beliefs and self-regulatory processes. From this standpoint, researchers have examined the association between achievement motivation and growth mindset. Empirical findings reveal that growth mindset is positively linked to MS but negatively linked to MF (Zhao et al., 2022). Zhao et al. (2024) posited growth mindset fosters an adaptive interpretation of failure, framing it as an opportunity for reflection and behavioural adjustment, experiencing higher intrinsic enjoyment and a sense of purpose when overcoming difficulties. This cognitive orientation enhances perseverance when facing setbacks and sustains effort towards long-term goals (Shi et al., 2023), thereby improving continuity and success in long-term goal pursuit (Du et al., 2023).

**Figure 1:**
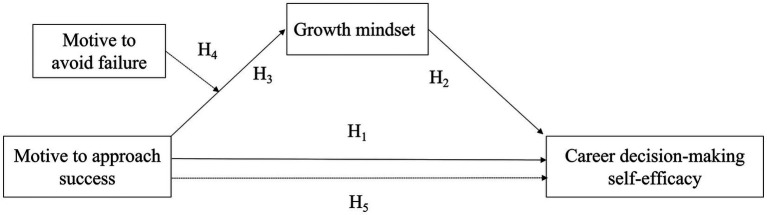
Proposed research model.

By contrast, individuals with weak achievement goals or low achievement motivation tend to exhibit lower psychological resilience (Zhan et al., 2024). When MF beliefs are strong, failures are more likely to be construed as evidence of inadequate ability, reducing perseverance and sustained effort in challenging situations (Birr et al., 2024). Collectively, these previous study results reveal that MS fosters a growth-oriented cognitive framework, whereas MF tends to hinder this cognitive restructuring, potentially impairing career confidence via its effect on growth mindset. Based on these theoretical and empirical considerations, this research suggests a research model illustrated in Figure 1. In this model, MS acts as a primary exogenous variable and positively predicting growth mindset, while MF is a factor moderating the effect of MS. Accordingly, the following hypotheses are proposed:

*H3*: MS significantly and positively predicts growth mindset.

*H4*: MF moderates the relationship between MS and growth mindset.

*H5*: Growth mindset mediates the relationship between MS and CDMSE.

*H6*: MF moderates the indirect effect of MS on CDMSE via growth mindset.

## 2 Methods

### 2.1 Sample

The current research utilized a quantitative method with a cross-sectional design. Participants were recruited using convenience sampling through the Credamo application, and comprised pre-service teachers from multiple public universities across a Chinese province, representing all 4 years of study. Data collection took place between March and July 2025. Standardized, structured questionnaires were administered online during class sessions under the supervision of trained research personnel, who provided participants with necessary instructions throughout the process. A total of 857 questionnaires were collected, and after removing invalid responses, 807 valid cases were retained. Participants had a mean age of 19.94 years, comprising 170 males (21.07%) and 637 females (78.93%). The questionnaire included sociodemographic information alongside three psychometrically validated scales.

### 2.2 Measures

#### 2.2.1 Motive to approach success and motive to avoid failure

Motive to approach success (MS) and motive to avoid failure (MF) were assessed using the Achievement Motivation Scale (Ye and Hagtvet, 1992). This scale comprises two subscales corresponding to the two components of achievement motivation. The MS subscale contains 15 items, designed to measure individuals’ perseverance in challenging tasks and their pursuit of personal excellence (e.g., “I am most attracted to jobs for which I am unsure I can succeed”). The MF subscale also comprises 15 items, assessing anxiety about failure and fear of difficulty when facing challenging tasks (e.g., “I fear failure in tasks that can only be completed given certain opportunities”). All items are rated on a four-point Likert scale ranging from 1 (strongly disagree) to 4 (strongly agree).

#### 2.2.2 Growth mindset

The Growth Mindset Scale (Chen et al., 2023) was used to assess individuals’ growth mindset. It consists of 18 items across six dimensions: (1) motivation, the intrinsic drive to learn (e.g., “My success mainly results from hard work and realizing my potential”); (2) attitude, beliefs about intelligence and effort (e.g., “As long as I study diligently, I can improve my intelligence”); (3) challenge, mindset when facing difficulties (e.g., “I am confident that I can persist and resolve problems effectively”); (4) grit, perseverance and diligence (e.g., “Effort will lead to positive outcomes and unexpected gains”); (5) adversity, coping with setbacks (e.g., “When my peers succeed, I analyze the reasons and learn from them”); and (6) positive mindset, confidence and openness (e.g., “I believe my intellectual ability is not fixed”). All items were positively coded, except for those measuring positive mindset, which were reverse-coded. Responses were rated on a 4-point Likert scale (1 = strongly disagree, 4 = strongly agree).

#### 2.2.3 Career decision-making self-efficacy

Career decision-making self-efficacy (CDMSE) was gauged using the brief Career Decision Self-Efficacy Scale (Fu, 2015), which comprises 19 items assessing individual perceptions across three domains: (1) career planning and career problem solving (e.g., “Select a profession that matches your preferred way of living”); (2) information gathering (e.g., “Persist in pursuing your professional or career goals even when facing setbacks”); and (3) career self-assessment (e.g., “Make career decisions without worrying about whether they are right or wrong”). For each item, participants indicate how confident they feel in their ability to perform that task. Responses are made on a five-point Likert scale, ranging from 1 (no confidence at all) to 5 (complete confidence).

### 2.3 Analysis strategy

The present study employed Partial Least Squares Structural Equation Modeling (PLS-SEM) using SmartPLS 4.0. We selected PLS-SEM over covariance-based SEM (CB-SEM) due to the following considerations: First, our theoretical framework involves a complex structural model, featuring both mediation (growth mindset) and moderation (motive to avoid failure) effects. PLS-SEM is superior in handling such complex path models, allowing for simultaneous estimation of multiple latent constructs and paths (Hair et al., 2019). Second, our study is prediction-oriented, seeking to confirm the predictive power of achievement motives on career decision-making self-efficacy. PLS-SEM is specifically designed to maximize the variance explained (R^2^) in target variables and assess predictive relevance, which is highly consistent with our confirmatory research goals.

Following established PLS-SEM procedures (Hair and Alamer, 2022), the reflective measurement model was evaluated in terms of reliability and validity. Reliability was examined using Cronbach’s alpha and composite reliability, convergent validity through loadings and AVE, and discriminant validity via the Fornell–Larcker criterion and HTMT.

Following Preacher and Hayes (2008), the structural model was estimated to evaluate both mediation and moderation effects. Mediation was inferred from the relative significance of direct and indirect paths when the mediator was incorporated into the model. Moderation was examined through the interaction effect between the focal predictor and the moderating variable.

## 3 Results

### 3.1 Reliability and validity of the measurement instruments

The reflective measurement model was evaluated using SmartPLS 4.0 in accordance with established PLS-SEM guidelines (Hair et al., 2019). Reliability and convergent validity were examined based on indicator loadings, composite reliability (CR), and average variance extracted (AVE), as reported in Table 1. Following recommended criteria, indicators with loadings below 0.40 were removed, whereas items with loadings between 0.40 and 0.70 were retained only when their inclusion did not adversely affect CR and AVE values. After this procedure, all retained indicators exhibited loadings above 0.573.

**Table 1:** Correlation, reliability, and validity analyses of measurement instruments.

Construct	*α*	CR	AVE	Fornell–Larcker Criterion				HTMT results			
				1	2	3	4	1	2	3	4
1. *MS*	0.93	0.94	0.53	**0.73**	–	–	–	–	–	–	–
2. *MF*	0.95	0.96	0.59	−0.27^**^	**0.77**	–	–	0.28	–	–	–
3. *GM*	0.95	0.95	0.59	0.56^**^	−0.25^**^	**0.77**	–	0.58	0.24	–	–
4. CDMSE	0.98	0.98	0.72	0.52^**^	−0.29^**^	0.56^**^	**0.85**	0.58	0.30	0.58	–
5. *MS* × *MF*								0.10	0.11	0.11	0.06
*M*				38.09	38.50	3.14	3.01				
SD				8.31	8.81	0.63	0.91				

Values in bold font are the square root of average variance extracted. Values under the diagonal are Pearson correlation coefficients with the Fornell–Larcker criterion applied. MS, motive to approach success; MF, motive to avoid failure; GM, growth mindset; CDMSE, career decision-making self-efficacy; CR, composite reliability; AVE, average variance extracted; HTMT, Heterotrait–Monotrait ratio. ^**^p < 0.01.

All items for motive to approach success (MS), motive to avoid failure (MF), and career decision-making self-efficacy (CDMSE) met the recommended thresholds and were therefore retained. In the growth mindset construct, four items representing the positive mindset dimension (GP1–GP4) demonstrated insufficient loadings and were excluded. Their removal resulted in an AVE exceeding 0.50 and CR values above 0.90, supporting adequate construct reliability and convergent validity.

Discriminant validity was further examined using both the Fornell–Larcker criterion and the Heterotrait–Monotrait ratio. For each construct, the square root of the AVE was greater than its inter-construct correlations, indicating adequate distinction among the latent variables. In addition, all HTMT values remained below the recommended threshold of 0.85. Taken together, these findings demonstrate that the measurement model exhibited satisfactory reliability and validity (Hair et al., 2012). The finalized model retained 15 indicators for MS, 15 for MF, 14 for growth mindset, and 19 for CDMSE.

### 3.2 Validation of the structural model

Table 2 presents the variance explained (*R*^2^) and predictive relevance (*Q*^2^) of the structural model. The R^2^ values indicate the model’s explanatory power, whereas the *Q*^2^values reflect its predictive relevance. Results showed that the exogenous variables accounted for 35.7% of the variance in growth mindset, while the model explained 37.5% of the variance in career decision-making self-efficacy (CDMSE). Furthermore, the cross-validated redundancy (*Q*^2^) values for growth mindset and CDMSE were above zero, indicating adequate predictive relevance of the structural model. According to the criteria proposed by Shmueli et al. (2016), these results indicate the model’s satisfactory predictive capability.

**Table 2:** Coefficient of determination (*R*^2^), *Q*^2^ and model fit (SRMR).

Endogenous latent factors	*R* ^2^	*Q* ^2^	SRMR
Growth mindset	0.357	0.207	0.051
Career decision-making self-efficacy	0.375	0.257	

Subsequently, Cohen’s *f*^2^ was employed to assess the relative contribution of the exogenous latent variables to the explained variance (*R*^2^) of the endogenous constructs (see Table 3). Following Cohen (1988), *f*^2^ values of 0.02, 0.15, and 0.35 represent small, medium, and large effects, respectively.

**Table 3:** Effect size (*f*^2^) and VIF evaluation.

Pathway	MS → GM	MS → CDMSE	GM → CDMSE	MF → GM	MS × MF → GM
*f* ^2^	0.428	0.095	0.171	0.021	0.047
Effect size	Strong	Weak	Moderate	Weak	Weak
VIF	1.086	1.463	1.463	1.089	1.017

VIF, Variance inflation factor.

The results indicated that, in predicting growth mindset, the motive to approach success (MS) exhibited a significant and large effect (*f*^2^ = 0.428), suggesting that it was the most critical predictor. In contrast, the motive to avoid failure (MF) showed only a small effect (*f*^2^ = 0.021). Additionally, the interaction term between MS and MF also demonstrated a relatively small effect (*f*^2^ = 0.047). In predicting CDMSE, growth mindset displayed a medium effect size (*f*^2^ = 0.171), indicating its considerable role in explaining variance in CDMSE. Conversely, the effect of MS on CDMSE was small (*f*^2^ = 0.095). Additionally, all variance inflation factor (VIF) values were below the conservative threshold of 3 (Hair et al., 2019), indicating that multicollinearity was not a concern.

### 3.3 Hypothesis testing

The PLS-SEM results (Table 4) indicated that the motive to approach success (MS) significantly and positively predicted career decision-making self-efficacy (CDMSE), supporting H1. Concurrently, MS significantly and positively predicted growth mindset, providing support for H3. Moreover, growth mindset exhibited a significant positive effect on CDMSE, supporting H2.

**Table 4:** Results of hypothesis testing.

Effect	Hypothesis and pathways	*β*	*t*	95% CI
Direct	H1: MS → CDMSE	0.295	7.302^***^	[0.216, 0.375]
	H2: GM → CDMSE	0.395	8.951^***^	[0.309, 0.482]
	H3: MS → GM	0.556	14.782^***^	[0.474, 0.619]
	MF → GM	−0.120	2.838^**^	[−0.203, −0.037]
	H4: MS × MF → GM	0.118	3.548^***^	[0.053, 0.183]
Indirect	H5: MS → GM → CDMSE	0.216	8.644^***^	[0.167, 0.265]

MS, motive to approach success; MF, motive to avoid failure; GM, growth mindset; CDMSE, career decision-making self-efficacy; CI, confidence interval.

^**^p < 0.01. ^***^p < 0.001.

Further analysis of the indirect effects revealed that growth mindset significantly mediated the relationship between MS and CDMSE, indicating that MS exerted a positive indirect influence on CDMSE via growth mindset. The mediating effect accounted for 42.27% of the total effect, supporting H_5_. Importantly, the interaction between MS and the motive to avoid failure (MF) significantly predicted growth mindset, demonstrating that MF positively moderated the relationship between MS and growth mindset. This finding confirms that MF strengthened the predictive effect of MS on growth mindset, thereby supporting H_4_.

**Figure 2:**
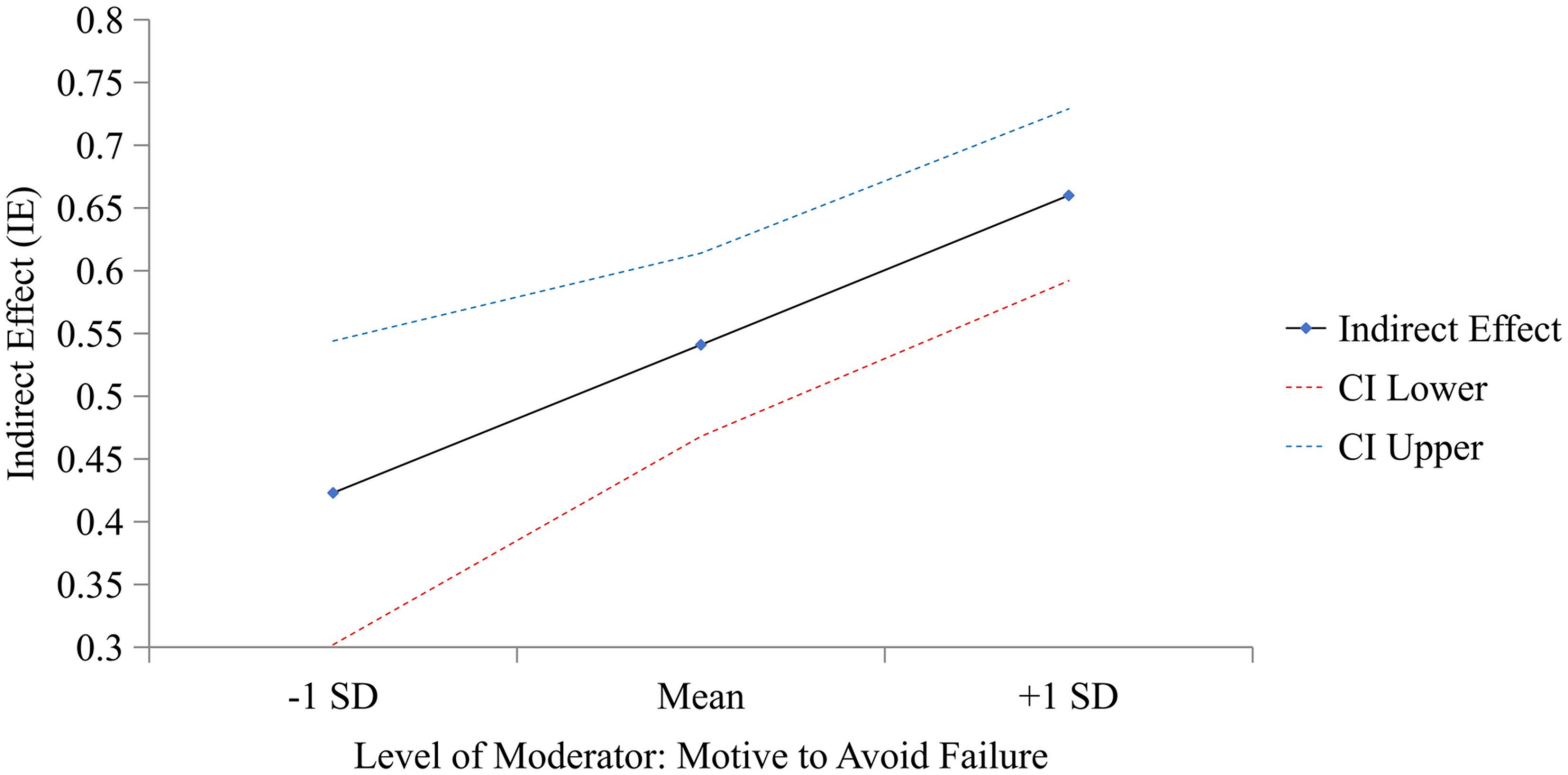
Conditional indirect effect of motive to avoid failure. LL, lower limit; UL, upper limit.

As shown in Table 5 and Figure 2, when MF is high, the predictive effect of MS on CDMSE via growth mindset is strengthened, thereby supporting H_6_.

**Table 5:** Moderated mediation analysis.

Path	Condition	*β*	*t*	95% CI
MS → GM conditional on MF	High MF (M + 1 SD)	0.660	18.892^***^	[0.592, 0.729]
	Average MF (M)	0.541	14.518^***^	[0.468, 0.614]
	Low MF (M − 1 SD)	0.423	6.845^***^	[0.302, 0.544]
MS → GM → CDMSE conditional on MF	High MF (M + 1 SD)	0.259	7.958^***^	[0.195, 0.322]
	Average MF (M)	0.212	8.527^***^	[0.163, 0.261]
	Low MF (M − 1 SD)	0.166	6.407^***^	[0.115, 0.216]
	Index of moderated mediation	0.047	2.983^**^	[0.016, 0.077]

CI, confidence interval. ^**^p < 0.01. ^***^p < 0.001.

## 4 Discussion

### 4.1 Motive to approach success and career decision-making self-efficacy

The analysing results of PLS-SEM confirmed that the motive to approach success (MS) of pre-service teachers positively predicts career decision-making self-efficacy (CDMSE). This finding is consistent with Lisá (2020), who highlighted achievement motivation as a critical predictor of career self-efficacy. Drawing on Social Cognitive Career Theory (Lent et al., 1994), high-MS individuals typically exhibit stronger career aspirations (Wang et al., 2022), which in turn predict greater career adaptability (Guan et al., 2016) and career decision confidence (Chen and Han, 2022).

Unlike individuals who experience career anxiety or low entrepreneurial intent due to fear of failure (Chua and Bedford, 2016; Gujral and Bhambri, 2024), pre-service teachers driven by MS maintain higher confidence in their career decisions even under potential risk of failure. The underlying mechanism appears to be that a strong achievement motive drives proactive behavioural engagement, enabling acquisition of essential career-related skills and resources (Li et al., 2022; Shi, 2022), while repeated practice and positive feedback accumulate professional experience, thereby reinforcing decision-making confidence (Wang et al., 2022). Consequently, pre-service teachers’ intrinsic drive to achieve in the educational domain translates into a firm belief in managing complex career choices successfully.

### 4.2 The mediating role of growth mindset

Mediation analyses revealed a key psychological pathway, whereby MS influences CDMSE through growth mindset. MS significantly predicted growth mindset, corroborating Zhao et al. (2024), they revealed a positive link between achievement motivation and growth-oriented thinking. A positive motivational orientation enhances adaptive capacity (Zhan et al., 2024), fostering the perception of challenges as opportunities for development instead of threats. The motivation-induced growth mindset serves as a catalyst for CDMSE (Park et al., 2022). As researchers noted, individuals with a growth mindset perceive their professional abilities as malleable and demonstrate persistence in learning despite setbacks (Burnette et al., 2020; Sik et al., 2024), thereby enabling more confident navigation of uncertainty in career decision (Li et al., 2025). Collectively, these findings suggest a significant association between MS and growth mindset, and indicate that growth mindset is positively linked to CDMSE. These patterns offer insights into how achievement motives may be related to career decision-making confidence through the potential mediating role of growth mindset, though further longitudinal research is required to establish causality.

### 4.3 The moderating role of motive to avoid failure

Traditional achievement motivation theories often treat MS and MF as functionally opposing systems: MS promotes engagement and belief in ability malleability, whereas MF is associated with threat perception and risk aversion, and is generally considered detrimental to growth mindset development (Atkinson, 1957; Covington, 1992). Previous research indicates that higher approach-oriented expectations are linked to growth mindset, whereas avoidance-oriented goals to fixed mindset and lower learning engagement (Heyder and Pegels, 2025).

However, the present findings offer a more nuanced perspective on these motivational functions. Moderation analysis revealed that, although MF is negatively correlated with growth mindset, it does not diminish the positive predictive effect of MS on growth mindset; rather, it significantly strengthens this relationship. Specifically, higher levels of MF enhance the extent to which MS translates into positive beliefs about the ability malleability. This suggests that, with high MF individuals, MS is not suppressed but further activated, directing cognition towards a more learning-oriented framework.

This aligns with theoretical advances in achievement motivation function reconstruction. Heckhausen and Heckhausen (2018) argue that the function of MF depends on its coexistence with MS: when MS is high, MF may serve an alerting and regulatory function rather than an inhibitory one, prompting allocation of greater cognitive resources in challenging situations. Similarly, Hobfoll (2011) posits that, under conditions of high perceived failure risk, individuals are inclined to develop sustainable psychological resources, for instance, growth mindset, to buffer against potential resource loss.

More specifically, this finding aligns with Compensatory Control Theory (Landau et al., 2015), which suggests that when individuals face threats to their sense of personal control—such as those induced by a motive to avoid failure—they become more motivated to embrace structured cognitive frameworks that restore predictability. In our study, the growth mindset potentially serves as such a framework, allowing individuals to mitigate the anxiety associated with MF by framing effort as a reliable mechanism for achieving success.

These results offer a nuanced perspective on the dual-motive dynamic. While MF typically impairs career confidence, our finding suggests that when high MS is present, MF may act as a ‘catalyst for urgency.’ Individuals high in both MS and MF face a greater perceived risk of failure, which appears to amplify their reliance on a growth mindset as a means to ensure performance success. This suggests that growth mindset functions as a buffer against the paralyzing effects of achievement anxiety, providing a pathway to maintain CDMSE even when the fear of failure is salient.

Overall, MF functions not as a negative moderator but as a context-dependent enhancer of the link between MS and growth mindset. This finding extends an explanatory scope of the dichotomous achievement motivation model, and provides a more dynamic, integrative perspective on the motivational foundations of growth mindset.

## 5 Conclusion and implications

The proposed model linked achievement motivation, growth mindset, and career decision-making self-efficacy (CDMSE), and was tested using PLS-SEM with data collected from pre-service teachers. The findings indicate that the motive to approach success (MS) significantly positive predicts both CDMSE and growth mindset. Growth mindset, in turn, significantly predicts CDMSE and mediates the relationship between MS and CDMSE. The findings further indicate a significant interaction between MS and MF. Specifically, the positive link between MS and growth mindset intensifies as MF increases. This pattern suggests that pre-service teachers who are strongly driven to achieve success are particularly likely to develop a growth-oriented belief system when accompanied by a heightened sensitivity to failure, ultimately fostering stronger confidence in career decision-making. Moreover, when both MS and MF are high, the positive influence of MS on growth mindset is further strengthened, thereby supporting enhanced CDMSE. These results highlight the dynamic interplay between motivational orientation and cognitive beliefs in shaping career-related confidence.

### 5.1 Practical implications

To help pre-service teachers navigate the uncertainties of future career choices more effectively, interventions should target both achievement motivation and cognitive beliefs. First, strategies aimed at encouraging MS could involves guiding students to set clear, specific, and attainable goals (e.g., SMART goals), enhancing outcome expectations and activating approach-oriented motivation. Breaking down long-term career objectives into manageable stages and providing ongoing monitoring might help frame career development as a gradual, growth-oriented process rather than a one-off high-stakes decision.

Second, MF could be addressed through functional guidance rather than simple suppression. Cognitive reframing interventions might reduce the tendency to equate failure with lack of ability, while encouraging interpreting failure concerns as cues for effort, preparation, and strategy adjustment. Our findings suggest that, under certain conditions, MF may be associated with growth mindset formation in a non-inhibitory manner.

Finally, learning and evaluation environments should emphasise ability malleability, the learning process, and individual progress rather than focusing solely on outcomes or social comparisons. Providing timely, specific, and improvement-oriented feedback allows pre-service teachers to accumulate mastery experiences and strengthen the confidence which abilities can be developed through effort, thereby cultivating a growth mindset.

### 5.2 Limitations and future directions

Notwithstanding its theoretical and practical implications, the present research is subject to certain limitations. Data were collected solely from pre-service teachers, which may restrict the generalizability of the findings and their transfer ability to different contexts. Replicating the proposed model in samples that encompass varied occupational and educational settings would help assess the stability of the observed relationships across subgroups. Second, subsequent studies could incorporate educational factors, for instance, school environment, family background, and individual characteristics, to explore how broader social and developmental contexts influence motivational processes and CDMSE.

In summary, this study provides empirical evidence of the relationships between achievement motivation, growth mindset, and career decision-making self-efficacy. Specifically, it suggests that the motive to approach success is linked to career decision-making confidence via growth mindset, and that the motive to avoid failure may function as a situational amplifier rather than a purely inhibitory factor. These insights offer avenues for designing interventions to enhance the career confidence of pre-service teachers.

## Data Availability

The raw data supporting the conclusions of this article will be made available by the authors, without undue reservation.
